# White Matter Repair After Extracellular Vesicles Administration in an Experimental Animal Model of Subcortical Stroke

**DOI:** 10.1038/srep44433

**Published:** 2017-03-16

**Authors:** Laura Otero-Ortega, Fernando Laso-García, María del Carmen Gómez-de Frutos, Berta Rodríguez-Frutos, Jorge Pascual-Guerra, Blanca Fuentes, Exuperio Díez-Tejedor, María Gutiérrez-Fernández

**Affiliations:** 1 Neuroscience and Cerebrovascular Research Laboratory, Department of Neurology and Stroke Center, La Paz University Hospital, Neuroscience Area of IdiPAZ Health Research Institute, Autonomous University of Madrid, Madrid, Spain

## Abstract

Mesenchymal stem cells have previously been shown to mediate brain repair after stroke; they secrete 50–100 nm complexes called extracellular vesicles (EVs), which could be responsible for provoking neurovascular repair and functional recovery. EVs have been observed by electron microscopy and NanoSight, and they contain associated proteins such as CD81 and Alix. This purified, homogeneous population of EVs was administered intravenously after subcortical stroke in rats. To evaluate the EVs effects, we studied the biodistribution, proteomics analysis, functional evaluation, lesion size, fiber tract integrity, axonal sprouting and white matter repair markers. We found that a single administration of EVs improved functional recovery, fiber tract integrity, axonal sprouting and white matter repair markers in an experimental animal model of subcortical stroke. EVs were found in the animals’ brain and peripheral organs after euthanasia. White matter integrity was in part restored by EVs administration mediated by molecular repair factors implicated in axonal sprouting, tract connectivity, remyelination and oligodendrogenesis. These findings are associated with improved functional recovery. This novel role for EVs presents a new perspective in the development of biologics for brain repair.

After stroke, not only is gray matter affected, but the white matter component is also compromised^1^,^2^. Given the complex pathways involved in the pathogenesis of brain damage, therapies aimed at more than one cellular target (i.e. not only neurons, but also oligodendrocytes, axons and myelin) might prove to be more efficacious. Mesenchymal stem cells (MSC) were extensively investigated for their reparative properties after stroke, MSC participate in processes such as neurogenesis, synaptogenesis^3^,^4^,^5^, oligodendrogenesis, axonal connectivity and myelin formation^5^, showing efficacy in not only gray matter affectation, but also white matter injury. The effect of MSC was related to a paracrine action^6^. This paracrine action might include soluble factors that are secreted into the extracellular environment^7^ that modify cell behavior in a paracrine manner favoring brain recovery.

Extracellular vesicles (EVs) are small heterogeneous microvesicles, 30 to 100 nm in diameter, that store within themselves multivesicular bodies (DNA, RNA, proteins and lipids) and information on their various biological functions and their cell type-specific molecular composition. They are widely distributed in serum, urine, saliva and other biological fluids. As important transfer vectors for intercellular communication and genetic material, EVs can stimulate target exerting their biological functions^8^ and might be responsible for the long-distance effects of stem cell therapy. Specifically, EVs derived from stem cells have recently been suggested to mediate restorative stem cell effects^9^ including being an interesting source for therapeutic applications in the field of regenerative medicine in neural disabilities including neurodegenerative diseases^9^,^10^ and stroke^7^,^11^. In particular, EVs induce long-term brain protection, promote gray matter repair and neurological recovery, and modulate peripheral post-stroke immune response^7^.

We aimed to investigate whether intravenous administration of MSC-derived EVs could induce functional recovery, promote oligodendrogenesis and aid white matter fiber repair when axonal tract integrity has been compromised, to potentially serve as a brain repair therapy after an experimental animal model of subcortical stroke.

## Results

### EVs characterization and biodistribution

**Figure 1:**
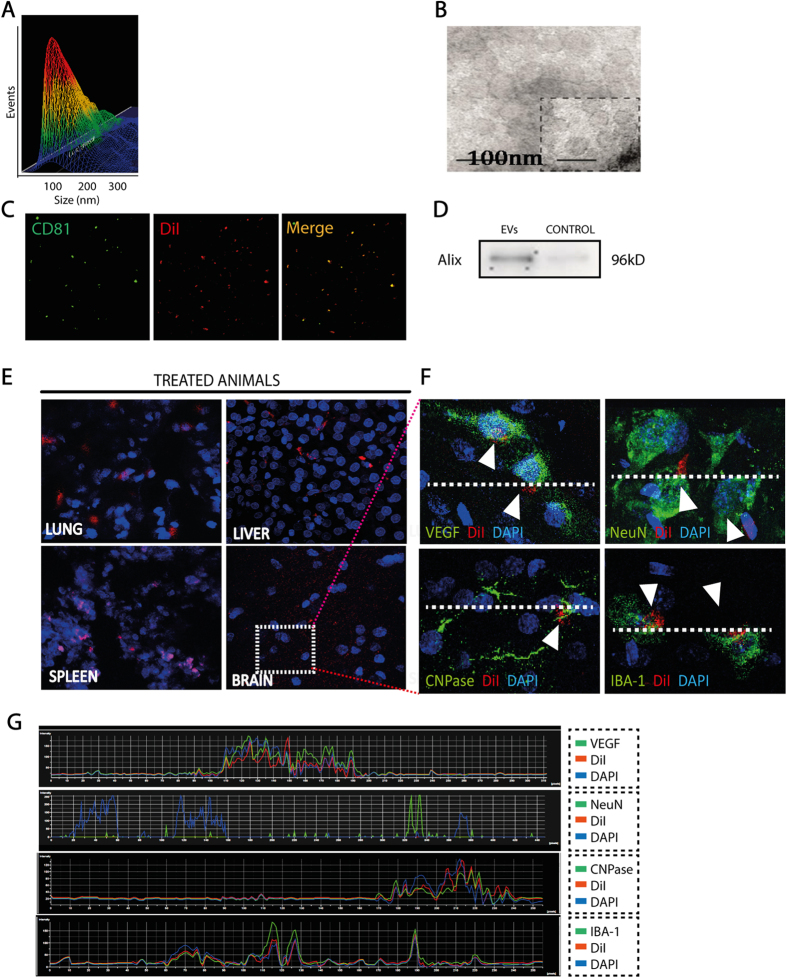
EVs characterization and biodistribution. EVs were characterized using various techniques (**A**) Histogram representing a distribution graph on a particle-by-particle (events) and size of the EVs using NanoSight (left), EVs image by NanoSight (right) (**B**) EVs image by electronic microscope, (**C**) immunoflorescence and (**D**) western blot. The gel image was cropped. (**E**) EVs (red) were found 24 hours after i.v. infusion in the brain, lung, liver and spleen by immunofluorescence. (**F**) DiI colocalization (representated by arrows) was shown with VEGF, NeuN, CNP-ase and Iba-1 in brain samples. White stroked line has been used to indicate the specific longitudinal section where the fluorescence intensity has been studied and represented in (**G**). (**G**) Quantification of fluorescence intensity of each marker found in the longitudinal profile of pixel of the white stroked line (n = 4 animals per group). Abbreviations: CNP-ase, 2′,3′-Cyclic-nucleotide 3′-phosphodiesterase; DAPI, 4′,6-diamidino-2-phenylindole; Iba-1, ionized calcium-binding adapter molecule 1; NeuN, Neuronal Nuclei; VEGF, vascular endothelial growth factor.

EVs have been observed to have typical morphology and size (<100 nm) by NanoSight (Fig. 1A) and electron microscopy (Fig. 1B). They contain associated proteins such as CD81 (a specific marker of EVs) and Alix using western blot and immunofluorescence (Fig. 1C and D).

DiI-labeled EVs were used for biodistribution and we analyzed histological sections of various organs. We found labeled EVs in brain tissue and in peripheral organs such as the lung, liver and spleen at 24 hours after administration (Fig. 1E). We also found co-labeling between DiI-labeled EVs with VEGF, NeuN, CNP-ase and Iba-1 at 24 hours after EVs administration (Fig. 1F and G).

### Effect of EVs treatment on functional recovery

**Figure 2:**
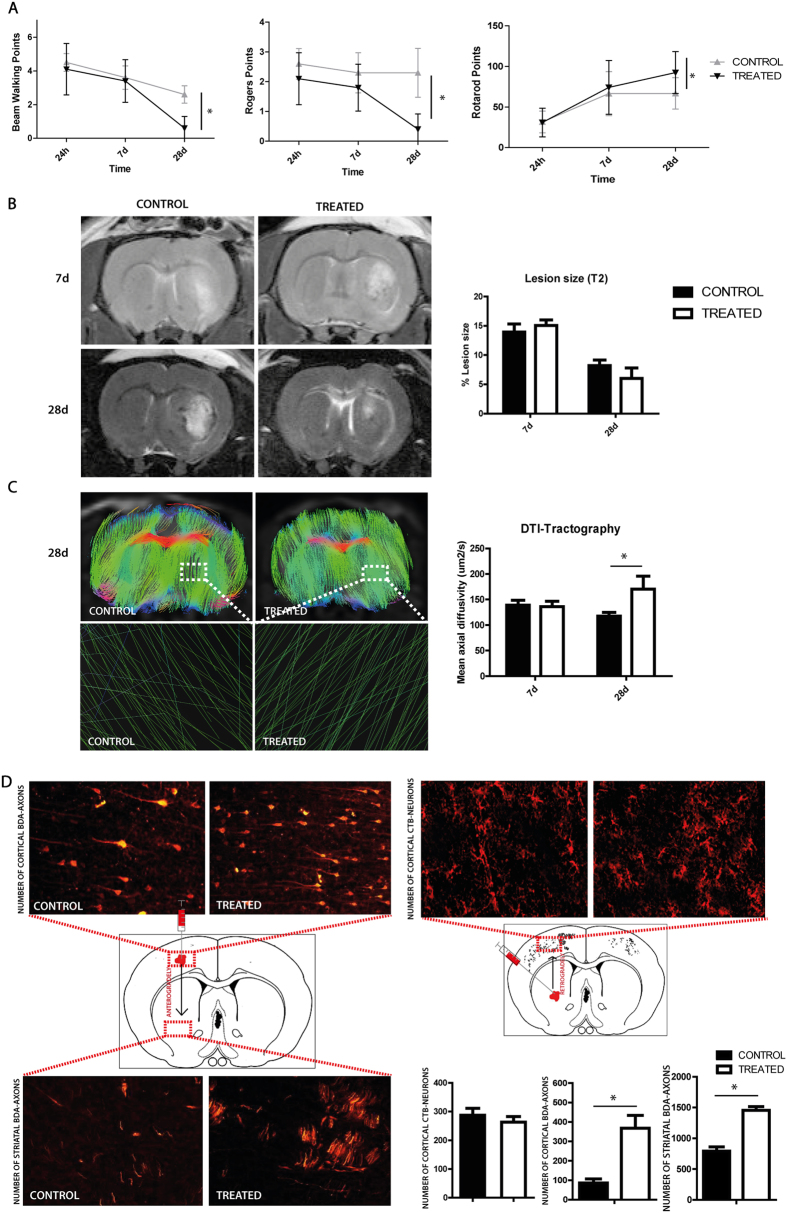
Improved functional outcome reduced infarct size and increased fiber tract and axonal sprouting after EVs treatment in subcortical stroke. (**A**) Beam walking test performance (left), the Rogers test (middle) and the Rotarod (right) was improved at 28 days (p < 0.05) in the treatment group compared with the controls (p < 0.05) (Data are shown as mean ± SEM, *p < 0.05; n = 10 animals per group). (**B**) Comparative image analysis of T2-weighted MRI at 7 and 28 days. (**C**) Comparative image analysis of tractography. Detail of tractography image in the lesion is given below at 28 days. Quantitative analysis of MRI images and tractography (data are shown as mean ± SEM, *p < 0.05, n = 4 animals). (**D**) Representative photomicrographs of cortical neuronal projections through the stroke site labeled with the anatomical tracer cholera toxin subunit B (CTB). Photomicrographs of cortical cells previously labeled with BDA and striatal axons from cortical cells labeled with biotinylated dextran amine (BDA). Quantification of cortical cells labeled with CTB that project to striatum (left), cortical cells labeled with BDA (middle) and striatal axons that have been projected from cortical cells labeled with BDA (right). (Data are shown as mean ± SEM, *p < 0.05, n = 4 animals). Abbreviations: BDA, biotin dextran amine; CTB, Cholera Toxin Subunit B.

No significant differences were found in the functional outcome of the treated and control animals at 24 hours and 7 days after treatment (p > 0.05). However, 28 days after treatment, the animals showed significantly better performance compared with the control group in beam walking (0.60 ± 0.699 vs. 2.60 ± 0.516, respectively; p < 0.001), the modified Rogers test (0.40 ± 0.516 vs. 2.30 ± 0.823, respectively; p < 0.001) and the rotarod test (92.40 ± 25.881 vs. 66.70 ± 19.322, respectively; p = 0.022) (Fig. 2A).

### Effect of EVs treatment on lesion size and tract connectivity

MRI analysis showed that there was not a significant difference between groups. The lesion size was indistinguishable in the treated animals compared with the control group at 7 days (15.06 ± 0.953 vs. 13.94 ± 1.364, respectively; p > 0.05). However, at 28 days, MRI-T2 results showed a tendency to decrease the lesion size in treated animals compared to control group (6.013 ± 1.788 vs. 8.21 ± 0.945, respectively; p > 0.05) (Fig. 2B).

DTI tractography data showed similar results for anisotropic fractions in both the controls and the treated groups 7 days after different types of stroke. However, 28 days after treatment, those rats receiving EVs showed significantly improved mean axial diffusivity compared with the control animals (170.47 ± 25.45 vs. 117.542 ± 7.255; p = 0.021) (Fig. 2C).

### Axonal sprouting after EVs treatment

White matter of the striatum was affected after subcortical ischemic stroke. To determine whether EVs could enhance the axonal sprouting process, the fascicles of the axons projecting from the overlying cortex were retrogradely labeled.

To make a baseline measurement of the severity of damage, CTB tracer was injected at the lesion site. CTB-labeled neurons were present in overlying cortical areas ipsilateral and contralateral to the stroke site. There was no difference in the severity of induced stroke between the treated and control animals, indicating that the white matter injury was similar in both (p > 0.05) (Fig. 2D).

Once we found that the severity of damage in white matter tracts was the same in both groups, axonal sprouting was studied using an anterograde neuronal tracer with BDA injected into the cortex. Three weeks after stroke, BDA was injected into the forelimb motor cortex because cortical projections have an important role in motor function and movement. Striatal axonal density was studied 1 week after the injection. A statistically significant number of BDA-labeled axons were shown in the striatum of the treated group compared with the control animals (367.91 ± 65.87 vs. 86.12 ± 21.17, respectively; p < 0.05). Moreover, we observed an increase of axonal density in the cortex area by anterograde BDA tracer in the treated group (791.32 ± 69.14 vs. 1456.32 ± 57.24, respectively; p < 0.05) (Fig. 2D). These results might indicate that EVs treatment produces significant axonal sprouting from the cortex to the striatum.

### Effect of EVs treatment on oligodendrocyte -associated markers expression

**Figure 3:**
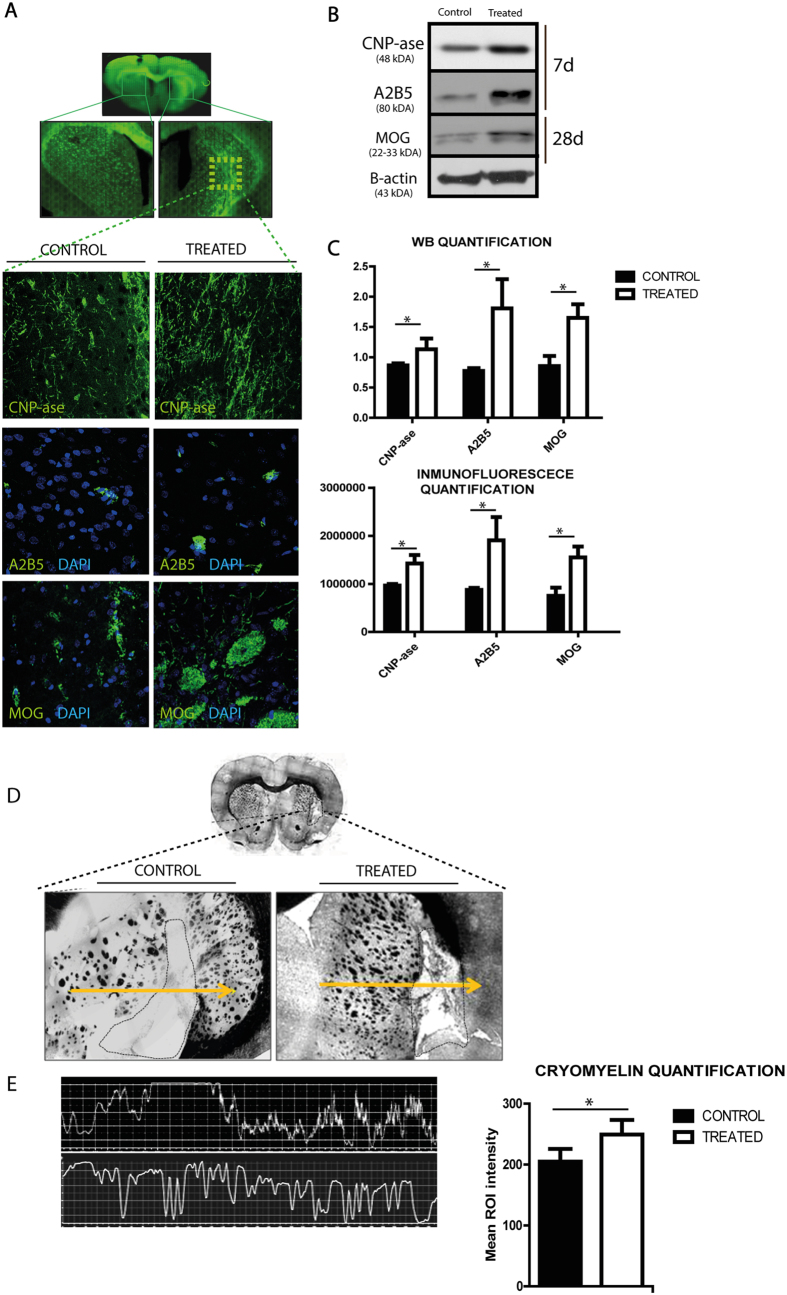
White matter-associated markers are enhanced after EVs therapy in subcortical stroke model (**A**) Immunofluorescence images of white matter repair-associated markers (CNP-ase, A2B5 and MOG). (**B**) Western blot images. The gels image was cropped. (**C**) Western blot and IF quantification of white matter repair-associated markers. (**D**) Morphological study by CryoMyelin staining identified the zone of the lesion as an area of white matter injury located in the subcortical zone, showing restored myelinated axons in the exosome-treated. Yellow line indicates a representative longitudinal profile of pixel intensity. (**E**) Quantification of the quantity of white colour in the along to the yellow line and quantification of mean ROI intensity of the CryoMyelin staining. (Data are shown as mean ± SEM, scale bars = 20 μm, *p < 0.05, n = 4 animals, 5 sections each per group). Abbreviations: CNP-ase, 2′,3′-Cyclic-nucleotide 3′-phosphodiesterase; MOG, Myelin Oligodendrocyte Glycoprotein.

Levels of markers related to the first steps of oligodendrocyte progenitor cell (OPC) development were analyzed in the lesion area 7 days after treatment (Fig. 3). CNP-ase appears to be one of the earliest events of oligodendrocyte differentiation. Notably, using western blot analysis, there was a significant increase in CNP-ase marker levels in EVs-treated animals compared with the control animals at 7 days after treatment (1.132 ± 0.177 vs. 0.865 ± 0.034, respectively; p = 0.05). (Fig. 3B and C). A2B5 is a cell surface ganglioside epitope expressed in OPCs, and its levels were higher in the EVs-treated animals compared with the control group at 7 days after treatment (1.807 ± 0.482 vs. 0.774 ± 0.045, respectively; p = 0.023) (Fig. 3B and C).

A marker related to later developmental steps in mature oligodendrocytes (MOG) was also analyzed at 28 days after treatment. After western blot analysis, there were significantly higher MOG levels in the animals that received EVs compared with the control group (1.650 ± 0.226 vs. 0.854 ± 0.167, respectively; p = 0.02) (Fig. 3B and C). All these results were confirmed by immunofluorescence analysis (Fig. 3A and C).

### Myelin restoration

Study of myelin fiber morphology identified the lesion zone in all the animals as a subcortical infarct affecting the white matter, with more myelinated axons in the EVs-treated animals compared with the control group (249.43 ± 3.90 vs. 204.78 ± 21.13, respectively; p = 0.032) (Fig. 3D and E).

### Proteomic analysis of the EVs

**Figure 4:**
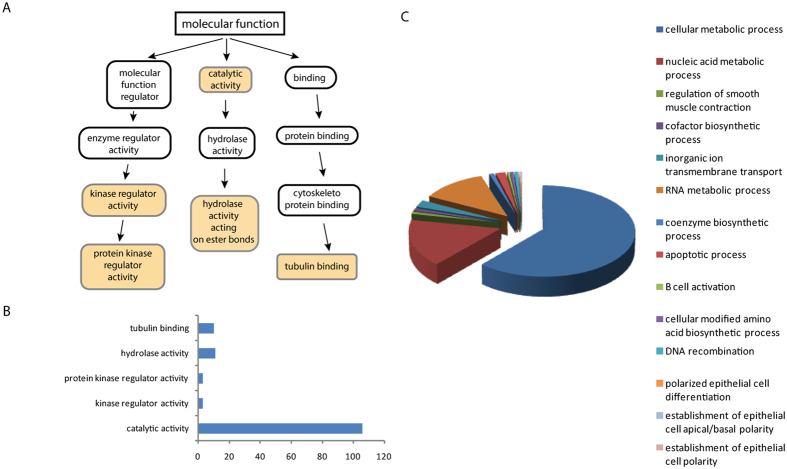
Proteomics analysis of ADMSC secretome reveals multiple biological functions (**A**) Gene ontology (GO) analysis of the 2,416 proteins identified by Orbitrab proteomic study. (**B**) Number of proteins associated with each biological function. (**C**) Processes in which are implicated proteins.

Proteomics analysis of the EVs identified 2416 proteins (Fig. 4) that are implicated in three different global functions such as molecular function regulator, catalytic activity and binding. All functions in detail are shown in Fig. 4A. The majority of the EVs-contained proteins are involved in hydrolase activity, tubulin binding, protein kinase regulator activity, kinase regulator activity and catalytic activity (Fig. 4B). Interestingly, the processes in which are implicated the proteins are cellular metabolic process, nucleic acid metabolic process, regulation of smooth muscle contraction, cofactor biosynthetic process, inorganic ion transmembrane transport, RNA metabolic process, coenzyme biosynthetic process, apoptotic process, B cell activation, cellular modified amino acid biosynthetic process, DNA recombination, polarized epithelial cell differentiation, establishment of epithelial cell apical/basal polarity and establishment of epithelial cell polarity (Fig. 4C).

## Discussion

The present study has demonstrated that a single administration of EVs is a therapeutic strategy to repair white matter damage after subcortical ischemic stroke. After intravenous infusion, EVs were found in the brain and in the peripheral organs (liver, lung and spleen). After EVs treatment, the animals showed improved functional recovery and increased axonal sprouting, oligodendrocyte-associated marker expression and myelin formation, white matter thickness (width, breadth, depth) and restoration of tract connectivity at 28 days. Proteomics analysis of the EVs identified 2416 proteins that are implicated in brain repair function.

The high frequency of white matter damage motivates the study of axonal sprouting and growth, oligodendrocyte formation, tract connectivity and remyelination because all are necessary for brain repair processes to improve functional and cognitive deficits after stroke. Several studies demonstrated that the administration of MSC improved functional deficits after various experimental models of stroke^12^,^13^. These beneficial effects were shown to be associated with a transient recruitment of MSC within the peripheral organs^4^,^5^. Based on this observation, it has been suggested that MSC might provide paracrine support for brain repair^14^.

EVs, are small vesicles released by cells bearing the surface antigen characteristic of the cell of origin. EVs might enter the target cells through specific receptor-ligand interactions and might deliver selected patterns of transferring receptors, proteins and bioactive lipids^9^. Thus, EVs could act as bioreactors, which could partially explain the paracrine effects observed in stem cell-based therapeutic approaches, and they can play a beneficial role in restoring tissue and organ damage. In the present study, EVs were found in both intracellular and extracellular compartments not only in the brain (area of injury) but also in various peripheral organs such as the lung, liver and spleen at 24 hours after treatment.

EVs purified from different cells have garnered tremendous interest based on the capacity of enhance repair in various pathologies of acute myocardial infarction^15^, chronic heart failure, spinal cord injury, stroke, wound healing^16^, cutaneous wound healing^17^ and proliferation of intestinal endothelial cells^18^. Even, EVs can act as an immune system regulatory tool^19^. The present study extends the effect of MSC-derived EVs to a model of subcortical ischemic stroke. In this model, EVs were used as a treatment to enhance white matter repair. Some studies have reported that GFP-tagged EVs enriched extracellular particles were released from MSC that were intravenously administered to rats with stroke and transferred to adjacent astrocytes and neurons^20^.

In a translational study it is important to analyze whether EVs treatment acts on the motor dysfunctions that are characteristic of subcortical stroke. In this study, EVs treatment induced a significant improvement in functional recovery that was particularly notable at 28 days after treatment compared with controls. These results clearly suggest a true recovery-enhancing effect from EVs. Although no previous studies have administered EVs in a subcortical stroke model, our results are consistent with previous data which demonstrated that systemic administration of MSC-generated EVs significantly improved functional recovery in other experimental animal model of stroke (ischemia) and traumatic brain injury^11^,^21^.

Recent studies demonstrate that intravenous administration of cell-free MSC-generated EVs enhances neurovascular plasticity and neurite remodeling in rats after stroke^22^. A subcortical stroke animal model was chosen to determine whether EVs treatment could enhance axonal sprouting and tract connectivity, given the region of the subcortical white matter contains fascicles of axons projecting from the overlying motor cortex. Neurons were labeled in an anterograde manner by a BDA injection into the cortex 3 weeks after stroke in order to map the connections growing after EVs treatment. A higher number of BDA-labeled axons were shown of the striatum in the treated group compared with the control animals. These results might indicate that EVs treatment produces a significant axonal sprouting response from the ipsilateral cortex to the injured striatum. All these results agree with those obtained by DTI tractography. This technique showed that fiber tract integrity and tract thickness was recovered at 28 days following EVs administration. These results suggest that good functional recovery at 28 days could be related to axonal sprouting white matter thickness (width, breadth, depth) and restoration of tract connectivity in the EVs-treated animals. For this reason, EVs administration could be involved in the process by which restructured axons, which had previously been compromised and demyelinated, recover not only their proper structure but also tract connectivity. These results are consistent with previous data showing neurite remodeling in the cortical ischemic boundary zone after other type of stroke^22^. Although there are no previous studies that study the effects of administered EVs in axonal sprouting after stroke, our results are complemented with previous data which demonstrated that EVs significantly increased the number of newborn mature neurons in the dentate gyrus after another type of neurology disease, traumatic brain injury^23^.

In this study, focal injection of the vasoconstrictor ET-1 into the subcortical white matter produced a visible infarct on MRI, as previously shown in subcortical stroke studies^24^,^25^. To analyze whether the EVs effect could be visualized with *in vivo* imaging techniques, we acquired T2-MRI maps. No significant differences in lesion size were observed in the EVs-treated animals compared with the control group on the T2-MRI images. These results agree with other studies that showed EVs-treatment did not reduce lesion size but significantly improved functional recovery after other type of neurological disease, traumatic brain injury^23^. Thus, the relationship, if any, between the lesion size and axonal sprouting and tract connectivity remained unclear. Thus we intensified the histological analysis of the injured tissue in this study.

For a correct brain repair process, not only axons, but also oligodendrocytes and myelin formation are important. In this study, we measured oligodendrocyte-associated markers in both the treated and the control animals to determine whether EVs administration also acts on oligodendrocytes and shattered white matter fibers. Related to the first steps in the genesis and migration of OPC, markers such as A2B5 (a characteristic OPC marker)^26^ and CNP-ase (markers related to earliest events in oligodendrocyte differentiation)^27^, were studied in the lesion area at 7 days after treatment. Markers related to white matter differentiation and myelin fiber maturation such as MOG were analyzed at 28 days.

In our study, the levels of the A2B5 and CNP-ase markers at 7 days and MOG at 28 days were higher in the cell-free EVs-treated animals. Although there have been no previous studies examining the effects of administered EVs in oligodendrocyte formation, our results are complemented with previous data that demonstrated systemic administration of EVs significantly reduced astrocytic GFAP expression and alleviated the expression of NeuN positive neurons after stroke in mice^28^.

Furthermore, in the process of white matter repair, the myelin sheath, which allows the transmission of nerve impulses over relatively long distances, is necessary for whole brain function. A cryomyelin study showed an increase of myelin in EVs-treated animals compared with the control group. These results could indicate that newly generated axons could themselves be covered by myelin.

In order to elucidate the mechanisms underlying all these repair processes the components of the EVs proteome need to be identified. Our proteomics analysis identified 2416 proteins; which are associated with several functions such as hydrolase activity, tubulin binding, protein kinase regulator activity, kinase regulator activity and catalytic activity. These results agree with other studies that revealed that secretome from MSC is related to protein binding and metabolic function^5^. Interestingly growth factor activity was not the main represented function in the cell secretome, indicating that other functions are also relevant. Our findings suggest that the identified proteins could contribute to improve functional recovery after EVs administration. When the specific molecules necessary for a therapeutic effect are known, selective manipulation of expression of those molecules might lead to an enhancement of the therapeutic efficiency of isolated EVs.

## Conclusions

In conclusion, our study helped us identify a clear role for EVs derived from MSC in improving functional outcome by mediating axonal sprouting and growth, oligodendrocyte formation, tract connectivity and remyelination after subcortical ischemic stroke. In the current study, EVs derived from MSC were found not only in the brain, but also in the peripheral organs, such as the lung, liver and spleen. Proteomic analyses identified 2416 proteins, some of which are associated with several regulation functions.

## Methods

### Ethics Statement

In this study, animal care and experimental procedures were strictly in accordance with the Guide for the Care and Use of Laboratory Animals, and approved by the Institutional medical school’s Ethical Committee for the Care and Use of Animals in Research of the La Paz University Hospital, according to the Spanish and European Union (EU) rules (86/609/CEE and RD53/2013). Experiments were reported according to all stroke therapy academic industry roundtable^29^ and RIGOR^30^ guidelines in terms of randomization, blinding and statistical powering.

### Animals and Surgery

The subcortical infarct (SCI) model has been well established^5^,^25^. Male Sprague-Dawley rats (200–250 g) were anesthetized using 3.5% isoflurane in 2 L/min oxygen and given meloxicam 2 mg/kg for analgesia. Subcortical stroke was induced by injection of 1 μL of endothelin-1 (ET-1; Calbiochem) (0.25 μg/μL) with the use of a SYR 5 uL Hamilton (Tecknokroma) into the striatum using stereotactic references (+0.4 mm AP, +3.5 mm L, +6 mm DV from bregma)

**Figure 5:**
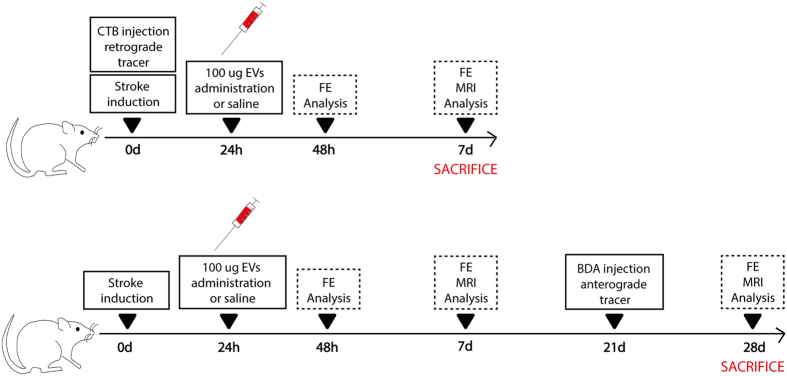
Experimental protocol schematic. EVs were isolated from rat adipose tissue-derived MSC and subsequently characterized. Male rats were injected with endothelin-1 to induce subcortical ischemic stroke. During the same surgery, half the animals were inoculated with CTB as Retrograde tracer. Twenty-four hours post stroke, treatment (saline or exosome) was administered through the tail vein. Later, 24 hours after treatment and on day 7, behavior and imaging and histological studies were evaluated. The other half of the animals were injected with BDA as anterograde tracer 21 days after treatment. Functional evaluation was studied at 48 hours, 7 and 28 days after treatment. These groups of animals were euthanized at 28 days. Abbreviations: BDA: biotin dextran amine; CTB, Cholera Toxin Subunit B; FE, Functional evaluation; MRI, Magnetic Resonance Image.

A total of 54 male Sprague-Dawley rats (8–9 weeks old, weighing 200–250 g) were used in this study. The study groups were as follows: (I) sham operation group (n = 10); (II) SCI control: SCI + 1 ml i.v. saline (n = 20); (III) SCI-treated: SCI + 100 μg i.v. EVs (n = 20) (Fig. 5). Additionally, 4 animals were used for biodistribution analysis, which were euthanized at 24 hours after treatment.

Treatment was administered by the tail vein at 24 hours after surgery. The rats were euthanized at 7 days or at 28 days (n = 10/each). One rat was excluded from the study because it died after stroke surgery.

### Cell culture protocol, isolation and EVs characterization

MSC obtained from allogeneic adipose tissue of Sprague-Dawley rats (250–300 g) were cultured. The adipose tissue was digested with collagenase (Sigma Aldrich) and incubated at 37 °C in 5% CO_2_. On the third pass, the cell cultures were characterized (CD90+/CD29+/CD45−/CD11b- cells) using a FACScalibur cytometer and CellQuest Pro software (Becton Dickinson). MSC for EVs isolation were cultured overnight with a free exosome-FBS culture medium. After 24 hours, cell supernatants were collected and the EVs were extracted using an exosome extraction kit (miRCURY^TM^ Exosome Isolation Kit, EXIQON), following the manufacturer’s instructions. EVs were used for treatment, for biodistribution study and for proteomic analysis and have been observed by electron microscopy and NanoSight. They contain associated proteins, such as CD81 and Alix using western blot and immunofluorescence.

### Biodistribution of EVs

The EVs were labeled with DiI (Celltracker CM-DiI, Invitrogen) prior to administration. Cryosections (40 μm thick) of lung, liver, spleen and brain were counterstained with 4′, 6-diamidino-2-phenylindole (DAPI), and the biodistribution was analyzed by immunofluorescence staining at 24 hours.

In order to characterize which cell type contains the EVs administered to the brain, we co-stained with DiI-labeled EVs and VEGF (1:500, Millipore), NeuN (1:100, Millipore), CNP-ase (1:500, Sigma-Aldrich) and Iba-1 (1:1000, Millipore), followed by the goat anti-mouse and anti-rabbit Alexa Fluor 488 antibody (1:750, Invitrogen). Images were acquired as a confocal maximum projection using a Leica TCS-SPE confocal microscope (Leica) and the number of double-positive cells was counted in a minimum of 6 different microscopic fields using a 40× objective lens and Image-ProPlus 4.1 software.

### Functional evaluation scales

Functional evaluation was performed for all animals by a blinded observer before surgery and after 48 hours, 7 days and 28 days. Motor performance was evaluated using the beam walking, Rogers and rotarod test. The beam walking test measured the ability of the rats to walk along a wooden beam (2.5 × 2.5 × 80 cm)^25^. The rotarod test measured the latency to fall from a rotating cylinder^5^. A variant of Rogers’ functional scale was used to assign scores^31^.

### *In vivo* magnetic resonance imaging (MRI)

Lesion size was analyzed after 7 and 28 days by MRI using a 7-Tesla horizontal bore magnet (Bruker Pharmascan, Ettlingen, Germany) and a T2-weight spin eco image. For this procedure, animals were anesthetized with a 2% isoflurante-oxygen mixture in an induction chamber and the flow of anesthetic gas was constantly regulated to maintain a breathing rate of 50 +/− 20 bpm. The lesion area was expressed as a percentage of the contralateral hemisphere, after correcting for brain edema. For tractography analysis, diffusion-weighted magnetic resonance images were acquired at 7 and 28 days after stroke. Diffusion tensor data were acquired with a spin echo single shot echo planar imaging (EPI) pulse sequence using the following parameters: TR/TE 8000/35 ms; a signal average of 12, 30 noncollinear diffusion gradient scheme with a diffusion weighting b = 500 s/mm^2^ and b = 1000 s/mm^2^, 18 slices with a slice thicknesof 1.5 mm without a gap, field of view 35 × 35 mm. Total imaging time was 1 h 44 min. All EPI data were acquired with a single shot EPI sequence, 96 × 96 matrix, and zero filled in k space to construct a 128 × 128 image matrix. Fractional anisotropy, mean diffusivity, trace, the eigenvalues and eigenvector maps were calculated with a homemade software application written in Matlab (R2007a) and the 3D fiber tract map was created using the MedINRIA DTI Track software (http://www-sop.inia.fr/asclepios/software/MedINRIA). Zoomed lesion site 3-dimensional diffusion tensor images were represented using ParaView 4.1.0 software as previously described^24^.

### Mapping of Motor Cortex Connections

An intracortical injection of 0.5 ul anterograde neuronal tracer biotinylated dextran amine ([BDA] 10.000 molecular weight, Life Technologies, Grand Island, NY, USA) was used to asses axonal sprouting after treatment using the following coordinates (x = 0.04; y = 0.35; z = 1.6). BDA was injected into the motor cortex of the rats at 3 weeks post treatment. The rats were euthanized 1 week after BDA injection. All the brains were stained with Cy-5-conjugated streptavidin (Jackson ImmunoResearch, West Grove, PA, USA). A DMLB epifluorescent microscope (Leica Microsystems) was used to observe sections under the Cy3 filter. The BDA cortical cells were quantified using neuroanatomical quantification software (StereoInvestigator, MBF Bioscience). Ipsilateral and contralateral axons of the striatum and cortex were studied^32^,^33^.

A retrograde neuronal tracer cholera toxin Subunit B (CTB) (Cy3-labeled CTb, List Biological Laboratories, Campbell, CA, USA, diluted to 20 mg/mL in 10% DMSO) was also used to analyze changes in axonal sprouting after treatment. For mapping of CTB axons, 0.5 uL of CTB (diluted to 20 mg/mL in 10% DMSO) was infused with endothelin-1. The animals were euthanized 7 days after treatment. Sections were mounted, cover slipped and visualized under the Cy3 filter on a DMLB epifluorescent microscope (Leica Microsystems). Cortical cells labeled with CTB were quantified using neuroanatomical quantification software (StereoInvestigator, MBF Bioscience)^32^,^33^.

### Immunohistochemistry, immunofluorescence and western blot analyses

Frozen sections were stained using the CryoMyelin Kit (Hitobiotech), which allows sensitive localization and visualization of myelin fibers. The mean intensity of myelin staining in the region of interest was quantified using a Nikon Eclipse-Ti inverted microscope and NIS-elements software. The lesion area was studied in detail using immunofluorescence and western blot analyses. The various antibodies used for these analyses were CNP-ase (1:500, Sigma-Aldrich), A2B5 (1:500, Millipore) and MOG (1:100, Abcam), followed by goat anti-mouse and anti-rabbit Alexa Fluor 488 (1:750, Invitrogen). For western blot analysis, the units were normalized based on Β-actin (1:400, Sigma-Aldrich). To quantify the expression of white matter-associated markers, the mean fluorescence intensity was evaluated using a 40× objective lens. The experiments, images and quantification of the samples were performed by blinded observers, using the same microscope configurations to eliminate bias due to background normalization (4 animals from each group).

### Proteomics data analysis

For proteomic analysis of exosome content, proteins were digested using the filter-aided sample preparation (FASP) protocol^34^. Briefly, samples were dissolved in 50 mM Tris-HCl (pH 8.5), 4% SDS and 50 mM DTT, boiled for 10 min and centrifuged. Protein concentration in the supernatant was measured by the Direct Detect^®^ Spectrometer (Millipore). Approximately 50 μg of protein was diluted in 8 M urea in 0.1 M Tris-HCl (pH 8.5) (UA), and loaded onto 30 kDa centrifugal filter devices (FASP Protein Digestion Kit, Expedeon, TN, USA). The denaturation buffer was replaced by washing 3 times with UA. The proteins were later alkylated using 50 mM iodoacetamide in UA for 20 min in the dark, and the excess alkylation reagents were eliminated by washing 3 times with UA and 3 additional times with 50 mM ammonium bicarbonate. Proteins were digested overnight at 37 °C with modified trypsin (Promega, Madison, WI, USA) in 50 mM ammonium bicarbonate at a 40:1 protein:trypsin (w/w) ratio. The resulting peptides were eluted by centrifugation with 50 mM ammonium bicarbonate (twice) and 0.5 M sodium chloride. Trifluoroacetic acid (TFA) was added to a final concentration of 1% and the peptides were finally desalted onto C18 Oasis-HLB cartridges and dried-down for further analysis.

Peptides were loaded into the LC-MS/MS system for online desalting onto C18 cartridges and analyzing by LC-MS/MS using a C-18 reversed phase nano-column (75 μm I.D. × 50 cm, 2 μm particle size, Acclaim PepMap RSLC, 100 C18; Thermo Fisher Scientific, Waltham, MA, USA) in a continuous acetonitrile gradient consisting of 0–30% B in 180 min, 50–90% B in 3 min (A = 0.5% formic acid; B = 90% acetonitrile, 0.5% formic acid). A flow rate of 200 nL/min was used to elute peptides from the RP nano-column to an emitter nanospray needle for real-time ionization and peptide fragmentation on an Orbitrap Elite mass spectrometer (Thermo Fisher). An enhanced FT-resolution spectrum (resolution = 35000), followed by the MS/MS spectra from the most intense 15 parent ions were analyzed along the chromatographic run. Dynamic exclusion was set at 30 s.

For peptide identification, all the spectra were analyzed with Proteome Discoverer (version 1.4.0.29, Thermo Fisher Scientific) using SEQUEST-HT (Thermo Fisher Scientific). For database searching at the Uniprot database, parameters were selected as follows: trypsin digestion with 2 maximum missed cleavage sites, precursor and fragment mass tolerances of 600 ppm and 0.02 Da, respectively, carbamidomethyl cysteine as fixed modification and methionine oxidation as dynamic modifications. Peptide identification was validated using the probability ratio method^34^ with an additional filtering for precursor mass tolerance of 10 ppm. The false discovery rate (FDR) was calculated using inverted databases and the refined method^35^. For the study of the biological functions of identified proteins, gene onthology analysis was performed using the GOrilla (Gene Ontology enRIchment anaLysis and visuaLizAtion) research tool^36^.

### Statistical analysis

Results were expressed as mean ± standard error (S.E.M.). Data were compared using the Kruskal-Wallis test followed by the Mann-Whitney test. Values of p < 0.05 were considered significant at a 95% confidence interval; the data were calculated using statistical SPSS 16 and GraphPad software.

## Additional Information

**How to cite this article:** Otero-Ortega, L. *et al*. White Matter Repair After Extracellular Vesicles Administration in an Experimental Animal Model of Subcortical Stroke. *Sci. Rep.* **7**, 44433; doi: 10.1038/srep44433 (2017).

**Publisher's note:** Springer Nature remains neutral with regard to jurisdictional claims in published maps and institutional affiliations.

## Supplementary Material

Supplementary Information
