# Supplementary material for: White Matter Repair After Extracellular Vesicles Administration in an Experimental Animal Model of Subcortical Stroke
Source: Sci Rep. 2017 Mar 16;7:44433. doi: 10.1038/srep44433 (PMC5353554; doi:10.1038/srep44433)
Supplement: Supplementary Information [file srep44433-s1.pdf]

White matter repair after extracellular vesicles administration in an experimental animal model of subcortical stroke

Laura Otero-Ortega; Fernando Laso-García*; María del Carmen Gómez de Frutos*; Berta Rodríguez-Frutos; Jorge Pascual Guerra; Blanca Fuentes; Exuperio Díez- Tejedor§; María Gutiérrez- Fernández§

[1]Neuroscience and Cerebrovascular Research Laboratory, Department of Neurology and Stroke Center, La Paz University Hospital, Neuroscience Area of IdiPAZ Health Research Institute, Autonomous University of Madrid, Madrid, Spain

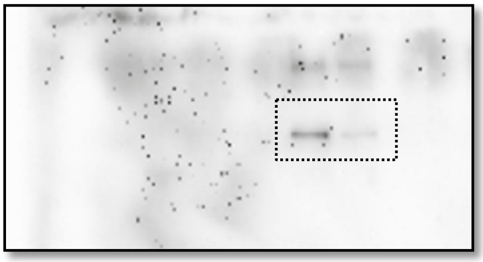

Figure S1: Uncropped and unprocessed original scans for all the blot in figure 1: EVs were characterized using Alix Marker by western blot**.** Stroked Square represents the zone of the blot used for Figure 1.

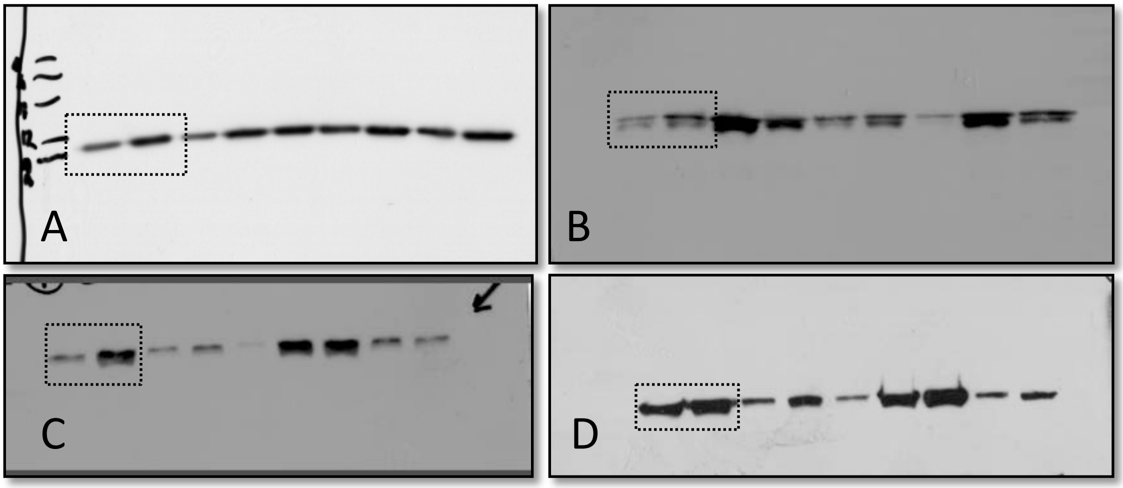

Figure S2: Uncropped and unprocessed original scans for all the blots in figure 3. White matter repair associated markers [CNP-ase marker.(A), MOG marker (B), A2B5 marker (C)] were studied in the lesion zone of the brain by Western blot. D) B-actin marker was used as loading control in Westner blot . Stroked Squares represent the zone of the blot used for Figure 3.
